# Assessment consistency in special care dentistry: an exploratory study of structured oral examinations

**DOI:** 10.3389/froh.2026.1863588

**Published:** 2026-07-21

**Authors:** Lisa Morlock, Sarah Rampf, Sebastián Véliz, Daniela Grothe, Philipp Linde, Benedikt C. Spies, Stefan Rupf, Jana Jünger, Anna-Lena Hillebrecht

**Affiliations:** 1 Department of Periodontology and Operative Dentistry, University Medical Center, Johannes Gutenberg University, University of Mainz, Mainz, Germany; 2 Department of Conservative Dentistry, Faculty of Medicine, Heidelberg University, University Hospital Heidelberg, Heidelberg, Germany; 3 Department of Periodontology, Dental Clinic, Sigmund Freud University, Vienna, Austria; 4 Faculty of Dentistry, University of Chile, Santiago, Chile; 5 Department of Prosthetic Dentistry, Center for Dental Medicine, Faculty of Medicine, Medical Center - University of Freiburg, University of Freiburg, Freiburg, Germany; 6 Department of Radiation Oncology, CyberKnife and Radiation Therapy, Faculty of Medicine and University Hospital of Cologne, University of Cologne, Cologne, Germany; 7 Synoptic Dentistry, Medical Faculty, Saarland University, Homburg/Saar, Germany; 8 CARES Institute, Heidelberg, Germany

**Keywords:** assessment consistency, competency-based assessment, dental education, known-groups validity, special care dentistry, structured oral examination

## Abstract

**Background:**

Assessing competencies in Special Care Dentistry (SCD) is challenging because domain experts are scarce and clinical decision-making is complex. Structured oral examinations (SOEs) may offer a standardized and scalable format for competency-based assessment.

**Objective:**

This study aimed to examine whether a SOE supports differentiation between predefined performance levels of SCD-related competencies.

**Methods:**

Twenty-four dentists without prior SCD experience independently rated two standardized, video-recorded mock performances representing different competence levels (high vs. moderate) based on the same clinical case. Ratings were performed using a five-point global rating scale (1 = excellent, 5 = fail) supported by a structured expectation guide. Descriptive statistics and inter-rater agreement were examined with particular emphasis on rating distributions and differentiation between performance levels (known-groups approach).

**Results:**

The high-quality performance was rated in the highest performance range by all examiners (23 ratings of 1, 1 rating of 2), whereas the moderate performance received lower ratings (13 ratings of 3; 11 ratings of 2). Rating distributions showed minimal variability and a clear separation between performance levels (median 1 vs. 3). Mean scores were 1.04 (SD 0.20) and 2.54 (SD 0.51), respectively. Although the intraclass correlation coefficient was low (ICC=−0.038), this most likely reflects the highly restricted variance inherent in the study design and does not negate the observed consistency of the rating patterns.

**Conclusion:**

This exploratory study suggests that structured oral examinations may support differentiation between predefined performance levels in SCD under standardized conditions.

## Introduction

Special Care Dentistry (SCD) encompasses oral healthcare for people with medical, cognitive, or functional conditions that require adapted clinical approaches and specialized professional competencies (1). Providing high-quality care in SCD requires not only technical proficiency, but also advanced skills in communication, risk assessment, ethical decision-making and interprofessional coordination (2). This aligns with contemporary competency-based medical education frameworks which emphasize observable performances and structured assessment (3). However, many dental schools face persistent challenges in teaching and assessing these competencies because domain experts are scarce and SCD cases are clinically complex (4). In addition, people with disabilities continue to face poorer access to dental care, and insufficient training of dental students in this area contributes to uncertainty in managing these patients (1, 5).

Traditional oral examinations are prone to variability, subjectivity, and the examiner's bias (6, 7). These issues are exacerbated in fields such as SCD, making assessing studentś performances particularly difficult due to the examiners' lack of experience in dealing with the specific needs of vulnerable patient groups (8). To ensure fair and defensible assessment decisions contemporary validity theory emphasizes interpreting and using assessment scores rather than focusing solely on the test format (9–11).

Contemporary validity theory conceptualizes validity as an argument supporting the interpretation and use of assessment scores, drawing on multiple sources of evidence rather than relying on psychometric indices alone (12).

Structured oral examinations (SOEs) are standardized formats that combine predefined case material, scripted examiner prompts, and anchored rating scales. In the present study, performance levels were represented by standardized mock performances (SMPs). Compared with unstructured oral examinations, this structured approach improves reliability, fairness, and transparency (6, 13, 14). The relevance of structured assessment in this field is further underscored by recent consensus-based learning objectives for dental students providing care for care-dependent older adults, which emphasize communication, supported decision-making, clinical management, and care-adapted treatment planning as core undergraduate competencies (15).

The controlled presentation of clinical complexity in SOEs enables the assessment of clinical reasoning, communication skills, and adaptive expertise, which are core competencies in SCD (16). Despite investigations of SOEs in several areas of health professions education, little evidence exists regarding their ability to produce reproducible judgements in SCD when examiners lack specific expertise.

Given the limited availability of SCD specialists, it is important to determine whether this assessment approach remains applicable when examinations are conducted by non-specialist raters. If examiners without specific SCD expertise can distinguish between predefined performance levels using a structured format, this would support broader implementation of SOEs in dental curricula and contribute to more equitable competency-based assessment (17). According to van der Vleuten the utility of an assessment is determined by its reliability, validity, feasibility, and educational impact (18).

The objective of this study was therefore to evaluate whether a structured oral examination supports differentiation between predefined performance levels of SCD-related competencies when conducted by examiners without prior expertise in SCD.

Specifically, we investigated whether non-specialist examiners could distinguish between standardized high and moderate levels of performance.

## Methods

### Study design

This exploratory video-based comparative assessment study employed a video-based comparative assessment design to examine whether dentists without prior expertise in Special Care Dentistry (SCD) could differentiate between contrasting levels of performance in a structured oral examination (SOE). Two standardized mock performances (SMPs) based on the same clinical case were independently rated by non-specialist examiners using a global rating scale. The study followed a known-groups validity approach, testing whether raters could distinguish between predefined levels of performance quality.

### Conceptual framework

The study was grounded in principles of competency-based assessment in health professions education. Structured oral examinations aim to reduce construct-irrelevant variance by standardizing case presentation, examiner prompts, rating criteria, and examination conditions. This is particularly relevant in SCD where access to domain experts is limited.

The examination blueprint was aligned with competency-oriented frameworks in dental education and informed by previously defined consensus-based learning objectives for the care of care-dependent older adults. Key domains included communication skills, the impact of medical and functional conditions on oral health, legal and care-related aspects, and clinical management.

### Development of standardized mock performances

Two SMPs were developed to represent contrasting levels of competence in prosthodontic care for an older patient with dementia and care dependency. Both performances were based on identical case material and examiner prompts and differed only in the quality of clinical reasoning and decision-making.

Scripts were blueprint-based and aligned with predefined anchor criteria derived from the National Competence-Based Learning Objectives Catalogue in Dentistry (19). The high-performing SMP demonstrated structured reasoning, patient-centred decision-making, appropriate risk appraisal, and explicit consideration of dementia-related limitations. It also included care-relevant strategies such as caregiver involvement and planning under real-world care conditions. The moderate-performing SMP was deliberately designed as a socially confident but clinically superficial performance characterized by vague goal setting, insufficient justification of treatment decisions, inadequate risk appraisal, and limited consideration of patient-specific factors. This contrast was intended to test whether raters would distinguish competence from presentation style. Scripts and case design were iteratively refined and reviewed by a group of five clinicians with expertise in prosthodontics and gerodontology, as well as two experts in medical education and assessment.

### Case vignette and examination task

The case vignette described an 85-year-old patient with mild dementia and care dependency who had lost his complete dentures and was accompanied by his daughter, who served as caregiver and legal guardian. Candidates were asked to recommend a treatment approach, justify therapeutic goals, identify risks, and discuss patient-related factors affecting treatment planning. In addition, they were required to outline the chronological treatment sequence and delegation of tasks within the dental team. The case was selected to reflect a clinically relevant SCD scenario requiring integration of technical, communicative, legal, and patient-centred competencies. The examination scenario was designed to reflect a typical Special Care Dentistry consultation in which clinical decision-making required consideration of the patient, the caregiver, and the legal context of care, including principles of patient participation and supported decision-making. No live patient examination, clinical performance assessment, or interpretation of additional diagnostic materials was included. All candidates received the same written case information and were assessed on their ability to integrate this information into an appropriate treatment recommendation and management plan.

### Standardization of examination delivery

Both performances were enacted by trained actors using scripted dialogue and recorded under identical conditions. Camera perspective, examiner prompts, and interaction structure were standardized across both videos. Both videos depicted the interaction between the examiner and the candidate only. The patient and caregiver were not physically present but were represented through the written case vignette provided to all raters. To disentangle communicative style from clinical competence the moderate-performing candidate was portrayed as confident and personable, whereas the higher-performing candidate appeared somewhat hesitant despite demonstrating superior clinical reasoning.

### Participants and rating procedure

Twenty-four dentists from 15 university-based dental departments across Germany participated as raters. They represented a broad range of dental disciplines, including prosthodontics, restorative dentistry, periodontology, oral and maxillofacial surgery, and orthodontics and were actively involved in teaching and examining dental students. Prior to participation, all raters confirmed that they had no formal postgraduate qualification, specialist training, or recognized focus in Special Care Dentistry or Gerodontology. The inclusion of examiners without specific SCD expertise was intentional and reflected the study objective of evaluating whether a highly structured oral examination could support consistent assessment in settings where SCD experts are scarce. No formal rater training or calibration was provided. Unlike calibration procedures, which typically involve benchmarking exercises, consensus discussions, and performance standard setting, raters received only a structured expectation horizon derived from the examination blueprint. This expectation horizon functioned as a standardized model answer based on predefined anchor criteria and outlined key domains such as treatment indication, risk appraisal, patient-centred decision-making, and consideration of dementia, care dependency, and caregiver involvement. Ratings were completed independently and without discussion among raters. The expectation horizon was intended to support competence-oriented judgments by directing attention to predefined performance criteria rather than subjective global impressions.

### Rating instrument

The rating was guided by a structured scoring framework that included predefined anchor criteria and an explicit expectation horizon (structured model answer). Performance was rated on a five-point global rating scale ranging from 1 = excellent to 5 = fail. A global rating scale was selected because it is considered particularly suitable for assessing integrated competencies such as clinical reasoning, communication, and decision-making and has been shown to outperform checklist-based approaches when evaluating higher-order clinical performance (20, 21). The expectation horizon translated the examination blueprint into concrete, assessable performance components and defined the level of detail and prioritization expected in high-quality responses. In line with established principles of structured oral examinations, the expectation horizon focused on the most relevant clinical and decision-making aspects rather than on an exhaustive list of possible answers, thereby supporting structured and competence-oriented judgments even among raters without specific expertise in Special Care Dentistry.

### Data analysis

Descriptive statistics (frequencies, means, medians, standard deviations) were calculated for each performance. Interrater reliability was assessed using the intraclass correlation coefficient (ICC). A two-way mixed-effects model with absolute agreement was applied to evaluate the consistency of ratings across raters. Both the reliability of single ratings [ICC (3,1)] and the reliability of the average ratings across all raters [ICC(3,k)] were calculated (22). In addition, 95% confidence intervals and corresponding F-tests were computed to assess the precision and statistical significance of the reliability estimates (23). Given the limited number of performances and the restricted variance, ICC results were interpreted cautiously. Greater emphasis was placed on rating distributions, separation between performance levels, and within-group variability. The analyses focused on whether raters consistently distinguished between predefined competence levels rather than on psychometric generalizability. For the statistical analysis IBM SPSS Statistics was used.

## Results

**Figure 1:**
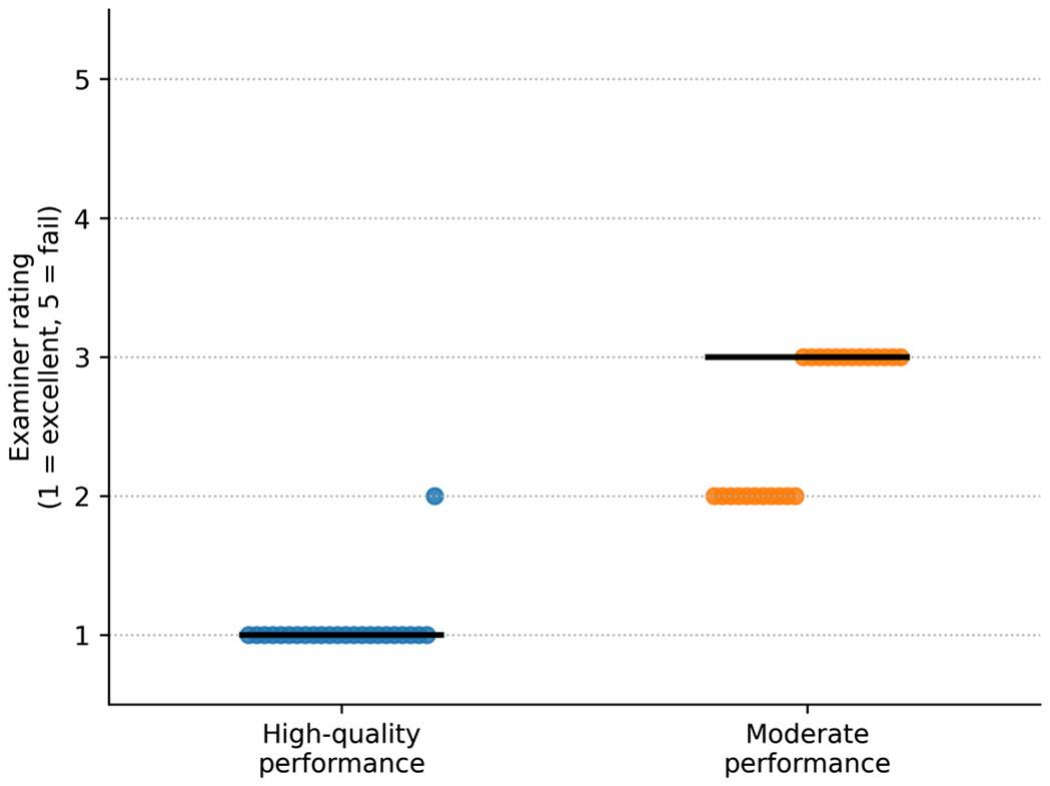
Figure 1 shows a scatter plot of examiner ratings for two standardized performances in a structured oral examination. Ratings were given on a five-point scale, with 1 indicating excellent performance and 5 indicating fail. The high-quality performance is clustered almost entirely at rating 1, with one rating at 2, whereas the moderate performance is clustered at ratings 2 and 3. Horizontal lines indicate the median ratings. The figure illustrates clear separation between the two predefined performance levels and limited variability within each rating group.

All 24 examiners completed the assessment of both performances. Ratings clearly distinguished the high-quality performance from the moderate performance across all raters. The high-quality performance was rated at the upper end of the scale by all examiners, whereas the moderate performance received distinctly lower ratings. Specifically, 23 examiners assigned grade 1 and 1 examiner assigned grade 2 to the high-quality performance, indicating that all ratings fell within the top performance range. In contrast, the moderate performance was rated as grade 3 by 13 examiners and grade 2 by 11 examiners (Figure 1).

These rating patterns demonstrate a clear separation between performance levels, with only minimal overlap in ratings (median 1 vs. 3). Ratings for the high-quality performance were clustered almost exclusively at the top of the scale, whereas ratings for the moderate performance were concentrated in the mid-range.

Descriptive statistics further underscore this distinction with a mean score of 1.04 (SD = 0.20) for the high-quality performance and 2.54 (SD = 0.51) for the moderate performance (Table 1). The relatively small standard deviations, particularly for the high-quality performance, indicate limited dispersion of ratings.

**Table 1:** Descriptive statistics of examiner ratings for the two standardized performances.

Performance level	Mean score	Median	SD
High-quality performance	1.04	1.00	0.204
Moderate performance	2.54	3.00	0.509

The distribution of individual ratings is illustrated in Figure 1 and visually reinforces the clear separation between the two performance levels.

Interrater reliability was assessed using the intraclass correlation coefficient (ICC) based on a two-way mixed-effects model with absolute agreement. The estimated agreement between raters was low, ICC (3,1) = −0.019, 95% confidence interval (CI) [−0.062, 0.078], F (23) = 0.729, *p* = .773. The reliability of the average ratings was similarly low, ICC (3,k) = −0.038, 95% CI [−0.133, 0.145], *p* = .773. However, these estimates should be interpreted with caution. The relatively small sample size and the resulting wide confidence intervals limit the precision of the reliability estimates. Furthermore, ICC values are highly sensitive to restricted variance and small numbers of rated cases. Given that only two standardized performances with minimal score dispersion were evaluated, variance-based reliability estimates were of limited interpretability. Therefore, the findings do not allow for firm conclusions regarding the true level of interrater agreement. The participating raters represented a heterogeneous sample from 15 academic sites and several dental disciplines. Given the exploratory study design and the small number of raters within each discipline, no discipline-specific analyses were performed. Importantly, all examiners, although not specialized in Special Care Dentistry, were qualified dentists and consistently differentiated between the two predefined performance levels.

## Discussion

This study demonstrates that structured oral examinations enabled examiners without specific SCD expertise to distinguish reliably between predefined performance levels under standardized conditions. The present findings should therefore be understood as providing targeted validity evidence for score interpretation under standardized conditions, particularly with regard to known-groups differentiation, rather than as a comprehensive validation of the SOE format across content areas and examination contexts (12).

These results are consistent with previous research showing that structured oral formats improve objectivity, fairness, and reliability compared with traditional oral (6, 16, 24). By standardizing case presentation, examiner prompts, and rating criteria, SOEs reduce examiner-dependent variability and support more transparent and standardized assessment decisions (25).

A key contribution of this study suggests that such structured formats may help reduce these effects and support more standardized evaluations, even among non-specialist raters. This distinction is important as structure cannot replace content expertise entirely, but it may help standardize judgment processes sufficiently to support comparable ratings in resource-constrained settings. This is particularly relevant in fields such as SCD, where access to trained specialists is often limited. Examiner judgments are known to be influenced by cognitive biases and variability in expertise, which may compromise reliability in unstructured assessment settings (26). The present findings suggest that structured formats can mitigate these effects and support consistent and standardized evaluations, even among non-specialist raters.

Likewise, the structured expectation horizon may have reduced variability by directing examiners' attention toward predefined competence criteria. Consequently, part of the observed consistency may reflect the structured scoring guidance rather than the SOE format itself, which should be considered when interpreting the findings. Global ratings have been shown to capture complex, integrated clinical competence more effectively than checklist-based approaches, particularly for higher-order skills such as clinical reasoning and communication (20, 21). In the present study, the combination of standardized case scenarios and global assessment appears to have facilitated consistent and well-aligned judgments across examiners.

From a broader educational perspective these findings align with contemporary frameworks of competency-based medical education, which emphasize structured, observable, and scalable approaches to assessment (3). Assessment utility framework, effective assessment formats must balance reliability, validity, feasibility, and educational impact (18, 27) Structured oral examinations appear well suited to fulfilling these criteria, particularly in resource-constrained settings in which expert examiners are scarce. From a medical education perspective, the relevance of these findings lies less in the specific clinical content of SCD than in the demonstration that assessment structure may support scalable and standardized judgments in small or emerging disciplines. Frenk et al. emphasize that high-quality examinations are essential for reflecting professional practice and for bridging the gap between education and healthcare (28). These considerations underline the importance of robust assessment approaches for preparing future dentists to care for vulnerable patient groups.

A notable finding of the present study was the low intraclass correlation coefficient (ICC), which would conventionally suggest poor inter-rater reliability. However, this result should be interpreted with caution. ICC estimates are strongly influenced by the amount of between-subject variance and may become unstable when the sample size is small and score variability is restricted. In the present study, only two standardized performances were evaluated, resulting in minimal variance between cases and a narrow distribution of ratings. Under such conditions, low or even negative ICC values may reflect statistical characteristics of the data rather than genuine disagreement among raters (22, 23).

Consequently, the ICC may not have been an optimal indicator of inter-rater reliability in this specific context. Although the coefficient did not provide evidence for high reliability, the observed rating patterns suggested a consistent differentiation between the two performance levels, with relatively little dispersion in ratings within each performance. These observations indicate that raters applied the assessment criteria in a broadly comparable manner, despite the low variance-based reliability estimates.

Nevertheless, the negative ICC remains an important limitation and prevents firm conclusions regarding the extent of inter-rater agreement. Future studies should include a larger number and broader range of performances to increase between-subject variability and allow for more robust reliability estimation. Such designs would provide a more suitable basis for evaluating inter-rater reliability using ICC-based approaches. Several limitations should be considered. First, the study included only two standardized performances under highly controlled conditions, which limits generalizability, restricts the range of observable variance, and provides only initial evidence for differentiation between predefined performance levels. Accordingly, the findings should not be interpreted as comprehensive validation of structured oral examinations in Special Care Dentistry. Although this design was appropriate for testing known-groups validity, future studies should include a broader spectrum of performance levels and multiple SCD scenarios. Second, the use of video-based assessments under controlled conditions may not fully capture the dynamics of live examination settings, in which interaction effects could influence examiners' judgment. In addition, the use of a single clinical case limits content validity and does not capture the full spectrum of SCD competencies. Structured oral examinations should therefore not be understood as stand-alone measures of competence but as one component within a broader program of assessment in SCD education. As a result, the study samples only a narrow portion of the construct domain, and conclusions should be limited to structured judgments in this specific assessment context rather than generalized to overall competence in SCD. Furthermore, ratings were based on standardized video performances rather than authentic examination encounters. Consequently, the findings may not fully transfer to live examination settings where examiner–candidate interactions are more dynamic. The deliberate contrast between one high-performing and one moderate-performing candidate may also have facilitated discrimination between performance levels. Future studies should therefore include a broader spectrum of performance quality. Although no formal rater calibration was performed, the structured expectation horizon itself may have reduced scoring variability by focusing examiner judgements on predefined assessment criteria. Therefore, part of the observed consistency may be attributable to the structured scoring guidance rather than to the SOE format itself.

Despite these limitations, the present study has important implications for educational practice. It suggests that structured oral examinations can serve as a scalable and resource-efficient tool for competency-based assessments in SCD. In line with recent proposals for implementing consensus-based gerodontology competencies into the German dental curriculum, SOEs may offer a practical way to translate such learning objectives into observable and assessable performances. By enabling more standardized assessment even in the absence of domain experts, SOEs may help expand assessment capacity, enhance inter-institutional comparability, and support the integration of SCD competencies into dental curricula. A particular strength of the design was the deliberate separation of communicative confidence from underlying competence, thereby addressing a well-documented source of bias in oral examinations, where presentation style may unduly influence judgments.

Ultimately, improving the assessment of SCD-related competencies may contribute not only to educational quality but also improve access to and quality of care for vulnerable patient populations, who continue to face substantial barriers to adequate dental services.

Our findings complement recent consensus work on undergraduate learning objectives for the care of dependent older adults. These competencies substantially overlap with internationally recognised domains of Special Care Dentistry, including communication, supported decision-making, legal considerations, and adaptation of treatment planning to functional and cognitive limitations. Consequently, the present assessment scenario may be viewed as representing core competencies relevant to both Gerodontology and Special Care Dentistry. Whereas the consensus study defined what future dentists should be able to do, the present study provides preliminary evidence that structured oral examinations may offer a feasible and scalable approach for assessing such competencies in a fair and standardized manner, even when domain experts are scarce (15).

Beyond its immediate educational implications, these findings support the systematization of competency-based assessment in SCD. Structured oral examinations offer a scalable solution that can be implemented across institutions, reducing dependence on scarce domain experts while maintaining consistent assessment standards. This may facilitate broader integration of SCD competencies into dental curricula and contribute to more equitable training conditions.

A central implication of this study is that structured assessment formats may partially decouple assessment quality from domain expertise, which is critical for scaling competency-based education in resource-constrained fields. This insight may be relevant not only for SCD, but also for other health professions education contexts facing similar challenges in assessment design.

## Conclusion

This exploratory study provides preliminary evidence that structured oral examinations may support differentiation between predefined performance levels in Special Care Dentistry under standardized conditions. As the study evaluated only two standardized performances, the findings should be regarded as initial evidence rather than a comprehensive validation of the assessment format. Future studies involving multiple cases, broader performance ranges, and authentic examination settings are required to establish generalizability and strengthen the validity argument.

## Data Availability

The original contributions presented in the study are included in the article/Supplementary Material, further inquiries can be directed to the corresponding author.
