# Supplementary material for: Assessment consistency in special care dentistry: an exploratory study of structured oral examinations
Source: Front Oral Health. 2026 Jul 21;7:1863588. doi: 10.3389/froh.2026.1863588 (PMC13433591; doi:10.3389/froh.2026.1863588)
Supplement: Supplementary file 1 [file Supplementaryfile1.docx]

**Supplementary Material S1**

**Structured Oral Examination (SMP)**

**Topic:**

Complete denture rehabilitation in a very old patient

**Discipline:**

Prosthodontics

**Subdiscipline:**

Gerodontology / Special Care Dentistry

**Evidence-based relevance / rationale:**

Access to dental care declines significantly in very old patients, particularly in individuals with dementia. Oral health is closely linked to nutrition, quality of life, and cognitive decline. Therefore, optimized prosthodontic care for older adults with care dependency represents a clinically and socially relevant competence area.

**Examination objectives:**

Indication and planning of complete denture therapy in care-dependent older adults.

**Assessment basis (competency framework):**

National Competence-Based Learning Objectives Catalogue in Dentistry (NKLZ), including:
- Indication and planning of removable prosthodontic treatments
- Realistic planning of treatment sequences

**Anchor criteria:**

Justifies indication for complete dentures in a patient-centred and evidence-based manner. Integrates functional, structural, and psychosocial aspects. Weighs treatment options (e.g., dentures vs. implants). Correctly describes and justifies the full chronological treatment sequence. Appropriately assigns tasks within the dental team.

**Case vignette**

An 85-year-old patient with mild dementia and care dependency (care level 2) has lost his complete dentures. He is accompanied by his daughter, who is his legal guardian and caregiver.

**Tasks**

1. Recommend a dental treatment, justify goals, risks, and patient-related factors.
2. Outline the treatment sequence and delegation within the dental team.

**Expectation horizon**

**Expected performance (excellent)**

Justifies the indication for complete dentures by integrating functional, structural, and psychosocial goals.

Weighs complete dentures against implant-based options and justifies a minimally invasive approach.

Identifies dementia-related risks, including impaired adaptation, reduced cooperation, and limited oral hygiene capacity.

Considers caregiver involvement in oral hygiene, appointment coordination, and prosthesis management.

Mentions additional risk-mitigation strategies such as duplicate dentures, denture labeling, or digital workflows.

Balances patient preferences, autonomy, and legal considerations while appropriately involving the caregiver in decision-making processes.

Describes the treatment sequence in the correct chronological order and assigns tasks to the dental team appropriately.

**Expected performance (satisfactory)**

• General indication correct
• Partial risk consideration
• Basic treatment sequence
• Limited patient-centred reasoning

**Performance leading to failure**

• No clear treatment indication
• No consideration of dementia/care dependency
• Incorrect or missing treatment sequence
• No justification of decisions
