# Supplementary material for: Assessment consistency in special care dentistry: an exploratory study of structured oral examinations
Source: Front Oral Health. 2026 Jul 21;7:1863588. doi: 10.3389/froh.2026.1863588 (PMC13433591; doi:10.3389/froh.2026.1863588)
Supplement: Supplementary file 2 [file Supplementaryfile2.docx]

**Supplementary Material S2**

**SMP Script 1 — High-performing candidate**

**Question 1: Recommend a dental treatment, justify goals, risks, and patient-related factors**

**Examiner:**
“Please recommend a dental treatment for this patient. Please also justify the therapeutic goals, relevant risks, and patient-related factors influencing your treatment planning.”

**Candidate (high-performing, content strong, slightly hesitant):**
“Thank you. In this case, I would recommend fabrication of new complete dentures.

The patient had previously worn complete dentures successfully on a daily basis, so this suggests that a complete denture rehabilitation is an appropriate and proportionate treatment option. I would therefore favour a minimally invasive approach.

Implant-supported treatment might theoretically be considered, but I would not recommend it here as the first option. The patient has mild dementia and care dependency, which may limit oral hygiene ability, cooperation, and long-term maintenance. In addition, he had already functioned well with conventional complete dentures before they were lost.”

**Examiner:**
“What treatment goals would you pursue?”

**Candidate:**
“The main goals would be to restore oral health-related quality of life. This includes improving masticatory function, supporting comfort and aesthetics, and enabling social participation.
In addition, complete denture rehabilitation may help to maintain oral function and may also contribute to dysphagia prevention. Another goal would be to preserve muscular and skeletal structures as far as possible.”

**Examiner:**
“What risks do you consider relevant in this case?”

**Candidate:**
“There are several important risks. First, due to the patient’s dementia, adaptation to a newly fabricated denture may be reduced.
Second, therapy and cooperation ability may be limited, which could affect the successful completion of all treatment steps.
Third, there is a relevant risk that the newly fabricated dentures may be lost again.”

**Examiner:**
“Which patient-related factors influence your treatment planning?”

**Candidate:**
“The most important patient-related factors are dementia, care dependency, and advanced age.

The dementia may affect oral hygiene ability as well as cooperation and treatment compliance.
The care dependency means that caregiver support is highly relevant, especially for appointment coordination, daily oral hygiene, and denture handling.
Advanced age may also indicate reduced resilience during treatment sessions, so appointments should be well structured and not unnecessarily demanding.”

**Examiner:**
“Would you consider any additional strategies?”

**Candidate:**
“Yes. I would involve the daughter closely, because caregiver support is essential in this situation.
In addition, I would consider denture labelling, duplicate dentures, and, where feasible, a digital workflow, because these strategies may reduce the consequences of renewed denture loss.”

**SMP Script 2 — Lower-performing candidate**

**Question 1: Recommend a dental treatment, justify goals, risks, and patient-related factors**

**Examiner:**
“Please recommend a dental treatment for this patient. Please also justify the therapeutic goals, relevant risks, and patient-related factors influencing your treatment planning.”

**Candidate (lower-performing, confident but superficial):**
“I would recommend making new complete dentures, because the patient does not have dentures anymore and obviously needs teeth again.

That would be the standard treatment here. Implants could also be considered, because they are more stable, but I think dentures would probably be easier.”

**Examiner:**
“What treatment goals would you pursue?”

**Candidate:**
“The goal is mainly that the patient can chew again and looks better.
It is also important that he is generally satisfied with the treatment.”

**Examiner:**
“What risks do you consider relevant in this case?”

**Candidate:**
“There are not many major risks with complete dentures. Maybe some pressure points at the beginning, but that is normal. Usually patients get used to them.”

**Examiner:**
“Which patient-related factors influence your treatment planning?”

**Candidate:**
“Well, he is older and has dementia, so treatment may be a bit more difficult.
But since the daughter is there, she can probably help with the practical things.
Overall, I would still say complete dentures are the best and simplest option.”

**Examiner:**
“Would you consider any additional strategies?”

**Candidate:**
“Not specifically. First I would just make the dentures and see how he manages.”
